# Hybrid cryptographic approach for strengthening IoT and 5G/B5G network security

**DOI:** 10.1038/s41598-025-21861-2

**Published:** 2025-10-30

**Authors:** Aman Kumar, Prashutosh Singh, Dhananjay Pandurang Kamble, Indrasen Singh

**Affiliations:** School of Electronics Engineering, Vellore Institute of Technology, Vellore, 632014 Tamil Nadu India

**Keywords:** Engineering, Mathematics and computing

## Abstract

The rapid evolution of fifth-generation (5G) and beyond (B5G) networks has introduced significant security challenges, necessitating advanced cryptographic mechanisms to protect sensitive data during transmission. Traditional encryption models often struggle to balance security, computational efficiency, and adaptability to dynamic network conditions. This study proposes a novel hybrid cryptographic framework integrating the Advanced Encryption Standard (AES), Data Encryption Standard (DES), and Rivest–Shamir–Adleman (RSA) algorithms. AES and DES provide high-speed symmetric encryption for efficient data protection, while RSA enables secure key exchange and authentication. The integration of dynamic round keys enhances encryption complexity, improving resistance to cryptanalytic attacks. Performance evaluations, including encryption and decryption time analysis, data expansion metrics, and throughput assessments, demonstrate that the proposed framework achieves an optimal balance between security and computational overhead. Benchmark comparisons with traditional and post-quantum cryptographic models highlight the superior efficiency and reduced data expansion of the hybrid approach. Furthermore, practical implementation on ESP32 hardware confirms the model’s feasibility for real-time encryption in resource-constrained environments typical of 5G applications. This scalable and flexible encryption paradigm addresses current and emerging security requirements in high-speed wireless networks, with future work focusing on integration with quantum-resistant cryptographic mechanisms to enhance resilience against evolving cyber threats. Experimental results show that the hybrid model achieves up to 30% higher throughput, 10–15% lower data expansion, and reduced encryption/decryption time compared to baseline algorithms, with successful ESP32 implementation and 100% decryption accuracy for key sizes up to 128 bits.

## Introduction

The emergence of Fifth-Generation (5G) and beyond (B5G) networks has led to a revolution in contemporary communication. It facilitates fast data transfer, rapid response times, and smooth connectivity between numerous devices^1^. Such attributes improve information sharing and enhance productivity in networks, but also pose enormous security challenges. Threats include unauthorized data access, spying, and sophisticated cyber threats such as MITM and DoS attacks. Moreover, the emergence of quantum computing raises concerns about how secure regular code-breaking techniques are given in^2–4^. Since 5G networks employ new technology such as network slicing and Multi-Access Edge Computing (MEC), traditional security tools are not effective enough for these distributed systems^5^. In hybrid Advanced Encryption Standard (AES) and Elliptic Curve Cryptography (ECC) propose cryptographic algorithm is proposed that ingeniously amalgamates the robustness of AES with the agility of ECC.

Regular code techniques, such as AES and RSA, secure data, but have a hard time maintaining security and computer resources. While AES encodes and decodes quickly, handling keys becomes complicated in large networks. Conversely, RSA is suitable for secure key exchange but requires much computer power, rendering it unsuitable to be used with real-time applications^6^. To resolve these issues, we require a new coding technique that’s highly secure and computer-friendly to suit the high-performance requirements of 5G and B5G networks^7^.

This research work proposes a hybrid cryptographic architecture that includes AES, DES, and RSA encryption to overcome the limitations of traditional encryption. The model proposed is quite effective in speeding up the process because AES and DES take advantage of high-speed symmetric encryption, while RSA is integrated with them for secure key exchange and authentication mechanisms. Dynamic round key generation further increases resistance against cryptanalysis and enhances the general security of time-critical 5G applications^8^. Designed with scalability and adaptability in mind, this framework can seamlessly integrate into future global wireless networks in response to the ever-changing security space of cloud computing, autonomous systems, and smart city infrastructures.

Crucial for most of the 5G challenges in security would entail proper key management, and this is especially where AES does not do well for secure key distribution on the one hand, while RSA is seen to place high demands on real-time applications with its processing speed. So, performance reasons and known threats make the asymmetric key-based model inconclusive. The work thus postulates, through hybrid symmetric and asymmetric encryption, that key management becomes feasible and efficient. This manages all the existing issues concerning the drawbacks in key exchange protocols^9^. The other consideration is a larger computational complexity in handling the security-sensitive applications that utilize an environment such as IoT and MEC. The proposed model can improve encryption efficiency without compromising security through the optimal scenario of combining AES, DES, and RSA, and is thus ready for latency-sensitive applications^10^.

The important attribute of the model is resistance against cryptanalysis, which is achieved through adaptive key scheduling and hybrid encryption mechanisms. Such enhancements seriously counter brute force differentials in cryptanalytic and auxiliary data attacks and thus give strong adaptability to the current evolution in cyberattacks^11^. On the other hand, this proposed cryptographic framework is intended to be extensible and flexible, fitting much wider applications outside normal network security, thus providing secure data transmission in fairly many applications, including various implementations of 5G infrastructure, autonomous vehicle communication, and industrial automation for completely secure coverage for new technologies^12,13^.

Quantum computing, the advent of new vulnerabilities with network slicing, and risks associated with edge computing challenge the security picture of 5G networks. Quantum computing will pose a grave threat to conventional encryption schemes such as RSA and ECC as these algorithms may be susceptible to quantum decryption attacks. To this end, several post-quantum cryptographic methods, including lattice-based encryption and CRYSTALS-Kyber, are currently under study intending to develop practical solutions^11^. Another example is that the very nature of network slicing-virtualized and isolated-introduces new security threats as attackers may breach inter-slice vulnerabilities. With an integrated encryption mechanism, the proposed model provides secure authorization and protected inter-slice communication as a way to mitigate possible attacks^12,13^.

There is an increase in performance, just as it increases the risk of potential cyberattacks because sensitive information is processed decentrally through the adoption of mobile edge computing. A well-defined encryption framework will guard data flow across edges and prevent malicious intrusion^14^. Modern encryption architectures such as homomorphic encryption, distributed ledger-based security frameworks, and AI-driven anomaly detection are used to boost security in 5G security frameworks today^15^. This hybrid cryptographic model developed in the paper is consistent with the trend and holds the promise of a robust, scalable, and efficient encryption strategy to fortify future communication networks against cyber threats and intrusions^16^.

### Related work

Because of the escalating complexity of cyber threats such as DoS attacks, quantum-based cryptanalysis, and unauthorized access, cryptographic security for 5G/B5G networks has become a major area of research. Various cryptographic techniques, namely symmetric, asymmetric, and hybrid encryption models, are being explored to enhance network security and data confidentiality. The integration of cryptographic algorithms allows secure communication, efficient key management, and scalability for modern network infrastructure^17^. Past work investigates architectural remedies by the 3rd Generation Partnership Project (3GPP) towards authentication enrichment of the 5G network. However, continually evolving threats push for added methods, like light cryptography and QKD towards the reinforcement of data integrity and authenticity^18,19^.

Many researchers have published lots of studies in relation to symmetric encryption techniques such as AES and DES for the purpose of securing real-time communication. Das et al. proposed an encoding-cryptography system using DES with an additional enhancement for security and including Hamming coding for error detection and correction in 5G networks^10^. It protects data from corruption and unauthorized access, thus increasing confidence in mobile communication. These types of symmetric encryption alone cannot satisfy key management challenges for large-scale networks, and they have to be combined with asymmetric cryptographic techniques to provide robustness in security.

Hybrid encryption models have been extensively studied to address these obstacles. Subedar and Araballi^11^ analyzed an encryption architecture that combines AES, RSA, and ECC in order to fend off brute-force threats and unauthorized access. Their study demonstrated that hybrid schemes trade computational efficiency for security, making these systems ideal for 5G and IoT ecosystems.

Likewise, Durge and Deshmukh^12^ proposed a hybrid encryption model integrated with cloud resources, which employs RSA and AES to achieve low-latency secure transmission with adaptive key generation. Whereas conventional cryptography schemes are interspersed with interest is growing in post-multiplication algorithms for securing infrastructures of 5G/B5G and systems. Sharma et al.^20^ implemented a quantum-safe security model for UAV communication in 5G networks, including AES, ECC, and CRYSTALS-Kyber. They stressed that future-oriented communication systems should immediately adopt quantum-safe key exchange. In addition, some of the research in WSN considered hybrid two-step cryptographic designs with fast encryption ensured by AES and key distribution secured by RSA to achieve both speed and protection^21,22^.

With the growing IoT and unprecedented levels of connected devices, cryptographic solutions need to be optimized in terms of low latency, high capacity, and efficient performance. One such stream cipher algorithm, Espresso, has been designed to offer both high-speed encryption and low hardware resource usage. Unlike conventional encryption mechanisms, Espresso presents a balanced security vs. computational efficiency trade-off, making it especially well-suited for systems with limited resources that operate in time-sensitive environments^23,24^.

Meanwhile, the hybridization of encryption with intelligent security frameworks serves to enforce zero-trust ideals, thus rendering further protection against evolving cyber threats. Although advances have been made, considerable research gaps remain, such as ensuring low-latency security solutions, lightweight cryptographic protocols, and a smooth transition onto next-generation networks. The scope of hybrid cryptographic methods presently suffers from computational overhead, scalability, and limited quantum-resistance^16,25^. To mitigate these disadvantages, this work proposes a novel hybrid framework of cryptography based on AES, RSA, and post-quantum cryptography, while simultaneously achieving security, efficiency, low latency, and adaptability in 5G/B5G environments.

### Motivation

**Figure 1:**
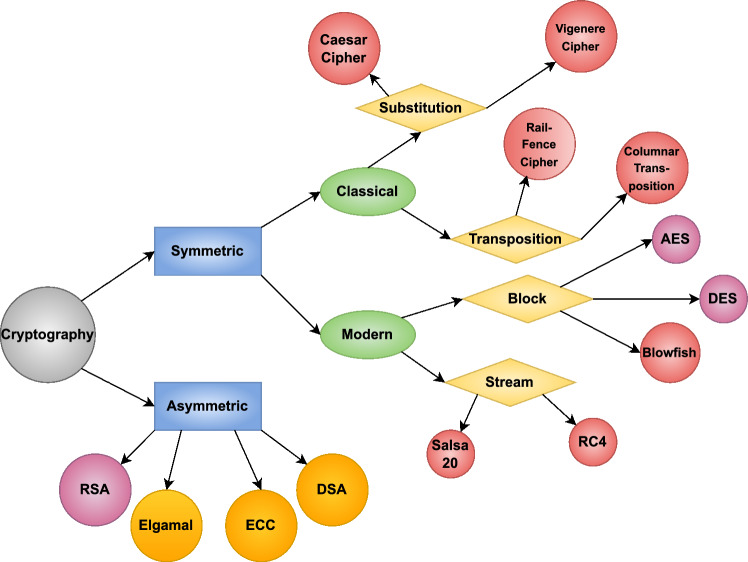
Different types of cryptographic techniques.

5G has witnessed rapid expansion at peak levels of establishing connectivity focused on enhancing real-time communications, IoT integration, and high-speed data transmission^26,27^. On the contrary, this has systematically increased the attack surface, making networks vulnerable to attacks such as MITM, unauthorized access, and data breaches. In 5G real-time applications, encryption algorithms like AES and RSA are considered impractical due to constraints that either involve threats to security or exorbitant computing costs. The aim of this research is to develop a hybrid model of cryptography that provides adequate security, practical encryption-decryption, and a scalable implementation for future networks^2^. Figure 1 Categorization of cryptographic techniques, such as symmetric and asymmetric encryption techniques, for communication security.

Researchers today highlight the vulnerabilities of stand-alone encryption algorithms to secure communication in 5G. AES deploys fast encryption but remains vulnerable to key distribution attacks. While RSA achieves strong authentication and integrity, it imposes substantial computational penalties^6^. Combining AES and RSA encryption techniques opens the door to optimization toward security with computational efficiency; hence, it is envisaged as a viable approach for securing smart cities, healthcare systems, and industrial IoT environments in 5G^8^.

This study seeks to impact in the sector of cybersecurity by enhancing hybrid cryptographic techniques for speed and security, enabling secure and real-time data transfer in 5G environments. The recommended hybrid model that includes AES-DES-RSA will not only be able to Optimize encryption-decryption performance but also tackle key management issues, that make it versatile for widespread deployment across secure cloud computing, autonomous vehicle communication, and encrypted 5G messaging^9,28^. By assessing the effectiveness of the AES-DES-RSA hybrid model regarding execution time, CPU load, and memory footprint, this study will contribute meaningful information to the real-world application of hybrid encryption schemes. The insights will help in designing more adaptive cryptographic platforms that is able to endure novel cyber risks associated with 5G and next-generation technologies^11,29^.

### Contributions

The major contributions of this work are summarized as follows:

- A novel hybrid cryptographic framework is proposed that integrates AES, DES, and RSA with dynamic round key generation, ensuring enhanced security and computational efficiency tailored to the high-speed and low-latency requirements of 5G and B5G networks.
- An extensive performance analysis has been conducted, including encryption/decryption time, throughput, and data expansion, demonstrating that the proposed model achieves superior efficiency compared to conventional and post-quantum schemes.
- The practicality of the framework has been validated through implementation on ESP32 hardware, confirming its suitability for resource-constrained IoT devices operating within high-speed 5G environments.

### Abbreviations

To maintain clarity and readability throughout this paper, the commonly used abbreviations and their meanings are listed in Table 1.

**Table 1:** List of abbreviations.

Abbreviation	Definition
3GPP	3rd Generation Partnership Project
5G	Fifth Generation
AES	Advanced Encryption Standard
B5G	Beyond 5G
CIEES	Communications Information Electronic and Energy Systems
CPU	Central Processing Unit
CRYSTALS	Cryptographic Suite for Algebraic Lattices
DDoS	Distributed Denial of Service
DES	Data Encryption Standard
DoS	Denial of Service
ECC	Elliptic Curve Cryptography
IoT	Internet of Things
ITNAC	International Telecommunication Networks and Applications Conference
MEC	Multi-Access Edge Computing
MITM	Man-in-the-Middle
PQC	Post-Quantum Cryptographic
QKD	Quantum Key Distribution
RSA	Rivest-Shamir-Adleman
S-box	Substitution-box
SDN	Software Defined Network
UAV	Unmanned Aerial Vehicle
WSN	Wireless Sensor Networks

### Organization of the paper

This paper has been organized as follows: first section provides an introduction and background on cryptographic techniques in 5G/B5G networks and stresses the need for a secure and efficient encryption scheme. Section “Proposed hybrid cryptographic model” Gives details about the proposed hybrid cryptographic model, by integration of AES, DES, and RSA cryptographic techniques, also describing the processes of encryption and decryption with security advantages. Section “Results and discussion” elaborate about results which show the performance evaluation of the proposed model concerning encryption efficiency and security strength, along with comparisons of existing cryptographic methods. Finally, “Conclusion and future work” discusses the conclusion and some of the main findings from this research. The outcome of the work suggests enhancements that will improve security and computational efficiency in the future.

## Proposed hybrid cryptographic model

The proposed hybrid encryption model employs a structured, multi-layered approach to enhance data security and encryption efficiency in 5G/B5G communication networks. Initially, the input data is converted into hexadecimal format and split into two equal parts: one half undergoes encryption using the modified Advanced Encryption Standard (AES), and the other half using the modified Data Encryption Standard (DES). Both AES and DES run for several rounds to make the encryption process more complex and secure. This parallel symmetric encryption process allows the model to benefit from the high speed and security of AES, alongside the simplicity and low computational cost of DES, thereby introducing a layered confusion mechanism and increasing resistance against linear and differential cryptanalysis.

**Figure 2:**
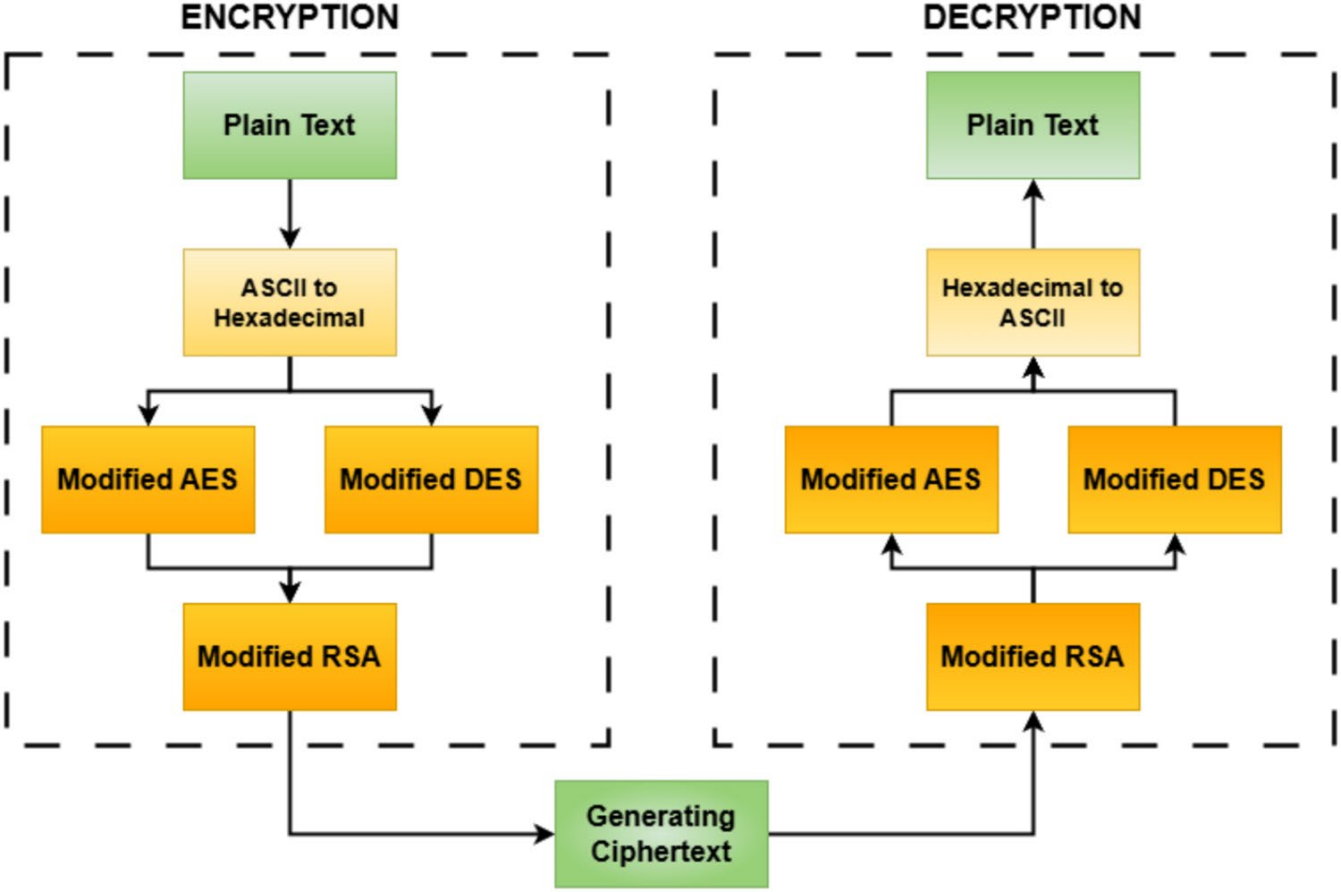
Block diagram of the proposed hybrid cryptographic model using modified AES, DES, and RSA for lightweight encryption in IoT and 5G/B5G systems.

Once the AES and DES encrypted outputs are generated, they are merged and further encrypted using Rivest-Shamir-Adleman (RSA), a public-key cryptosystem. RSA encryption secures the combined symmetric output, particularly useful for key distribution and safeguarding the integrity of the encrypted payload during transmission in open or untrusted networks. This hybrid strategy effectively combines speed (through symmetric encryption) and security of key exchange (through asymmetric encryption), making it robust against various attack vectors, including brute-force, replay, and man-in-the-middle attacks. Such a modular architecture is especially suited to the demands of 5G/B5G, where both performance and end-to-end security are critical. Figure 2 shows the Block diagram of the proposed Hybrid Cryptographic Model incorporating modified AES, DES, and RSA algorithms tailored for lightweight encryption in IoT and 5G/B5G environments.

### Encryption process

**Figure 3:**
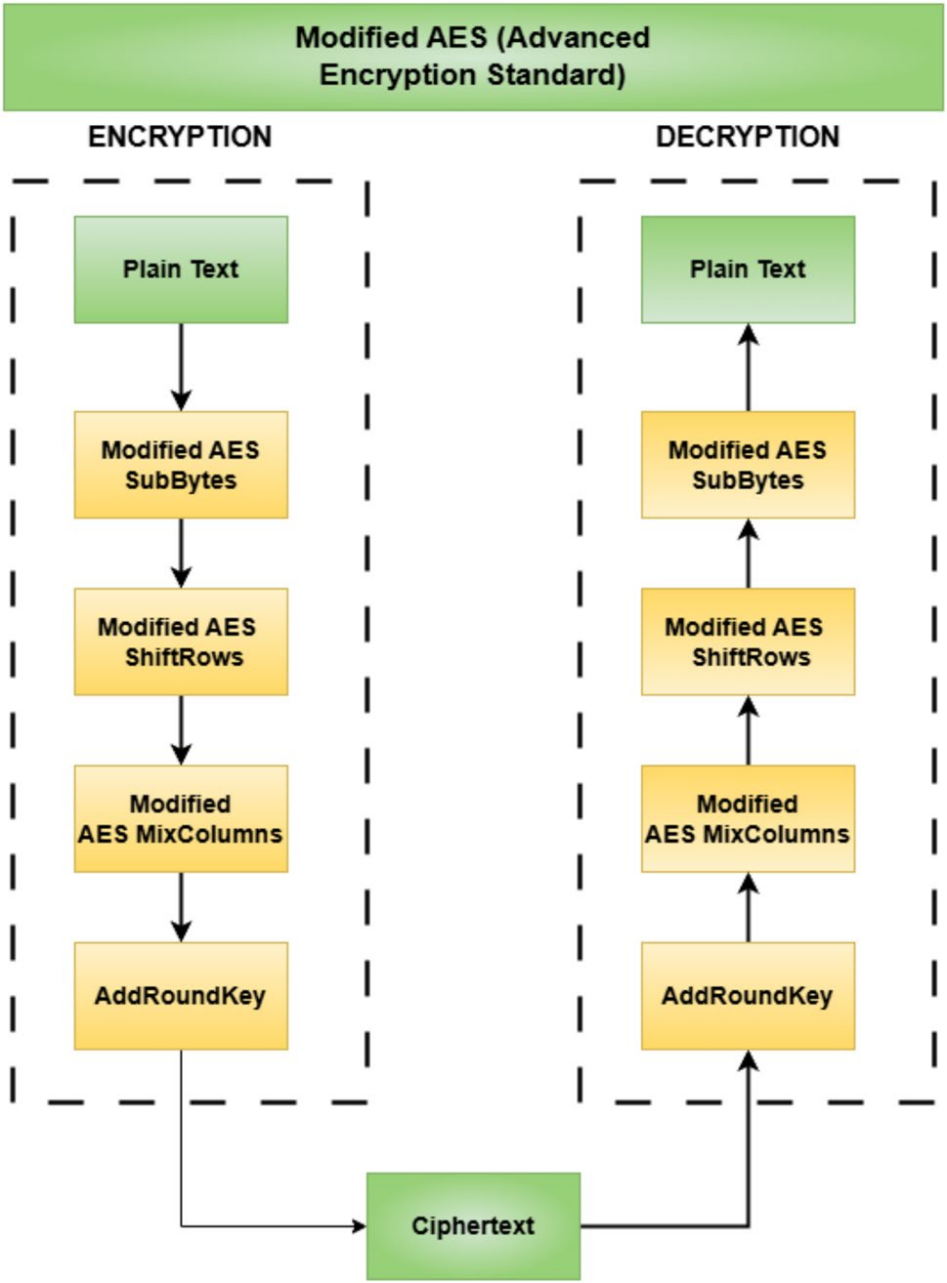
Block diagram of the modified AES algorithm.

The plaintext undergoes the process of splitting into two halves. Each half is encrypted independently for security purposes using modified AES and DES. In Modified AES, Fig. 3 shows the transformation procedure incorporates SubBytes, ShiftRows, MixColumns, and AddRoundKey operations, which are repeated for several rounds to secure the transformation of the data. On the other hand, it applies the Feistel network S-box substitution permutation functions, which will strengthen the encryption process in DES. When both halves have been encrypted, they are XORed using the dynamic round keys to add more complexity to the data. Lastly, it will be using RSA encryption for key exchange purposes to leverage the speed of symmetric encryption with the security of asymmetric encryption.

The S-Box substitution is an essential aspect of AES encryption that builds in non-linearity, enhancing the encryption’s resistance to attacks. Each byte in the plaintext is replaced by a byte defined in a substitution box or S-Box such that even the slightest change in the plaintext will most likely yield a different ciphertext. The modified AES S-Box consists of values arranged in a 4$\times$4 grid, ensuring complex transformations:

1 $$\begin{aligned} \text {S-box} = \begin{bmatrix} \texttt {0x64} & \texttt {0x25} & \texttt {0x3A} & \texttt {0xD0} \\ \texttt {0x7F} & \texttt {0x5D} & \texttt {0xF8} & \texttt {0x2B} \\ \texttt {0xA7} & \texttt {0xE1} & \texttt {0x4C} & \texttt {0x61} \\ \texttt {0xC3} & \texttt {0x1B} & \texttt {0x9E} & \texttt {0x87} \end{bmatrix} \end{aligned}$$

The ShiftRows operation then rearranges the bytes by shifting each row by a different offset, preventing a direct one-to-one mapping between input and output bytes. This step increases diffusion, making it harder for attackers to recognize patterns in the encrypted data. The updated ShiftRows transformation follows:

2 $$\begin{aligned} \text {ShiftRows} = \begin{bmatrix} a_{0,0} & a_{0,1} & a_{0,2} & a_{0,3} \\ a_{1,0} & a_{1,1} & a_{1,2} & a_{1,3} \\ a_{2,0} & a_{2,1} & a_{2,2} & a_{2,3} \\ a_{3,0} & a_{3,1} & a_{3,2} & a_{3,3} \end{bmatrix} \end{aligned}$$

MixColumns further strengthens diffusion by applying matrix multiplication in the Galois Field $GF(2^8)$. This process ensures that any change in one byte influences multiple bytes in the output, significantly increasing the complexity of the encryption. By spreading the impact of small changes across the entire block, MixColumns makes it much harder for attackers to identify patterns or reverse-engineer the encryption process.

3 $$\begin{aligned} \text {MixColumns} = \begin{bmatrix} \texttt {0x03} & \texttt {0x0B} & \texttt {0x09} & \texttt {0x0D} \\ \texttt {0x0D} & \texttt {0x03} & \texttt {0x0B} & \texttt {0x09} \\ \texttt {0x09} & \texttt {0x0D} & \texttt {0x03} & \texttt {0x0B} \\ \texttt {0x0B} & \texttt {0x09} & \texttt {0x0D} & \texttt {0x03} \end{bmatrix} \end{aligned}$$

Finally, the AddRoundKey step introduces key-dependent randomness by XORing the transformed data with a round key derived from the main encryption key. This ensures that even if the plaintext remains the same, different encryption keys will produce unique ciphertexts. The dependence on round keys adds an additional layer of security, making the encryption process more resistant to brute force and cryptographic attacks. The AddRoundKey transformation in AES is defined as follows:

4 $$\begin{aligned} \begin{bmatrix} b_{0,0} \oplus k_{0,0} & b_{0,1} \oplus k_{0,1} & b_{0,2} \oplus k_{0,2} & b_{0,3} \oplus k_{0,3} \\ b_{1,0} \oplus k_{1,0} & b_{1,1} \oplus k_{1,1} & b_{1,2} \oplus k_{1,2} & b_{1,3} \oplus k_{1,3} \\ b_{2,0} \oplus k_{2,0} & b_{2,1} \oplus k_{2,1} & b_{2,2} \oplus k_{2,2} & b_{2,3} \oplus k_{2,3} \\ b_{3,0} \oplus k_{3,0} & b_{3,1} \oplus k_{3,1} & b_{3,2} \oplus k_{3,2} & b_{3,3} \oplus k_{3,3} \end{bmatrix} \end{aligned}$$

where $b_{i,j}$ represents the state matrix and $k_{i,j}$ represents the round key.

**Figure 4:**
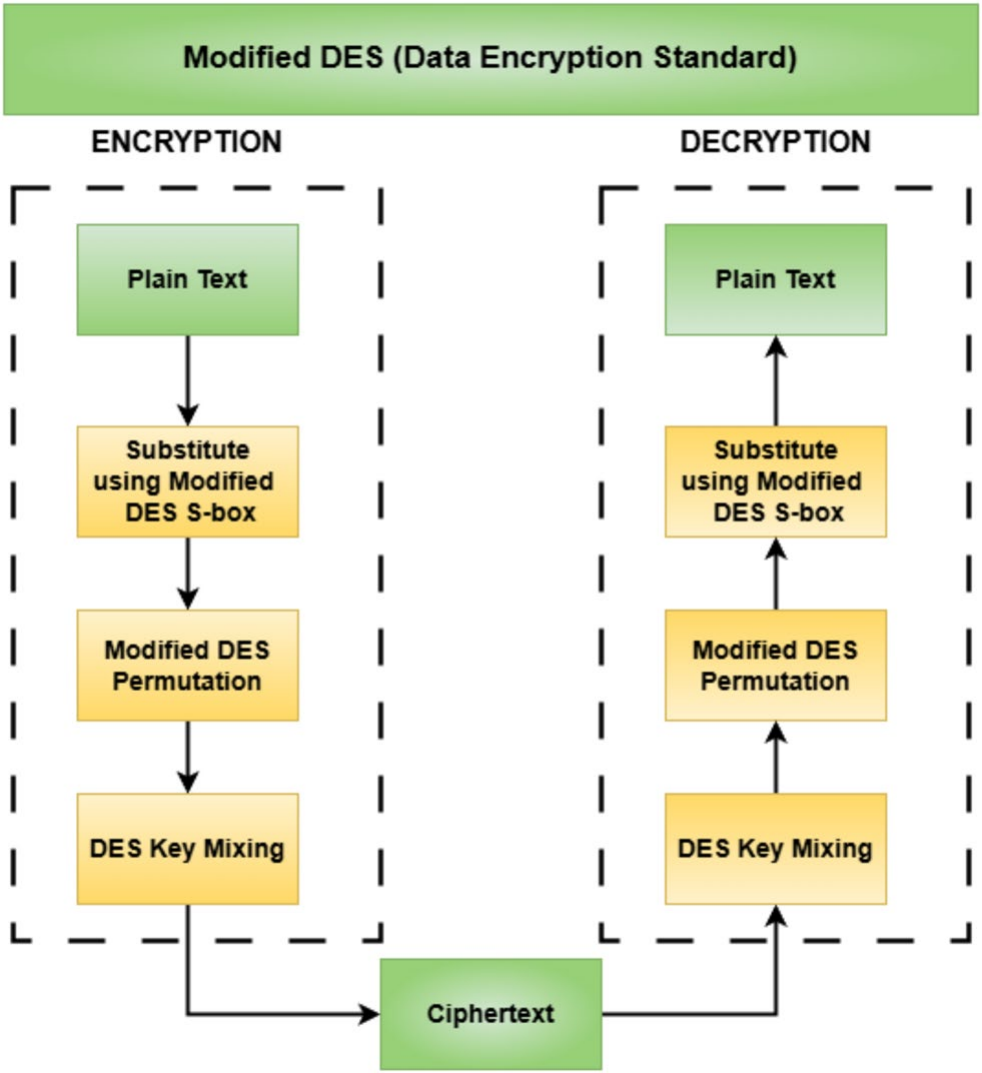
Block diagram of the modified DES algorithm.

The process in modified DES for 16-bit rather than the classical 64-bit blocks has been shown in Fig. 4. Two 16-bit halves are transformed by S-Box substitution, bitwise permutation, and key mixing. Specifically, during S-Box substitution, the 16-bit input is processed as two 8-bit segments, wherein each segment is mapped to a new value according to an S-Box definition.

This substitution provides the non-linearity, making it very challenging for an attacker to discover any pattern in the encryption scheme. The substitution is followed by a fixed permutation over another table that permutes the bits for diffusion purposes, thereby ensuring that even small changes to the input will produce drastic changes in the ciphertext, making cryptanalysis harder. The modified DES S-Box follows:

5 $$\begin{aligned} \text {S-box} = \begin{bmatrix} \texttt {0x2E} & \texttt {0x9C} & \texttt {0x61} & \texttt {0x18} \\ \texttt {0x73} & \texttt {0x4F} & \texttt {0x37} & \texttt {0x86} \\ \texttt {0xD5} & \texttt {0xA2} & \texttt {0xEF} & \texttt {0xC9} \\ \texttt {0xB1} & \texttt {0x08} & \texttt {0x50} & \texttt {0xAD} \end{bmatrix} \end{aligned}$$

After substitution, a fixed permutation table is applied, rearranging the bits to increase diffusion. This ensures that small changes in the input result in significant changes in the ciphertext, enhancing security against cryptanalysis. The updated permutation table for DES follows:

6 $$\begin{aligned} \text {Permutation table} = \begin{bmatrix} 16 & 29 & 1 & 5 \\ 7 & 12 & 15 & 18 \\ 20 & 28 & 23 & 31 \\ 21 & 17 & 26 & 10 \end{bmatrix} \end{aligned}$$

Thus, in this permutation step, the changed data is usually subjected to an exclusive-or operation with a unique round-specific key. The mixing of keys in this form guarantees that different outputs are acquired for subsequent rounds of encryption, such that no decryption could be done in the absence of the correct key. In the case of the combined AES-DES encryption method, the left half of the data passes through AES transformation while the right half goes for DES transformation. The halves are swapped at the end of each round, thus increasing the strength of encryption even further. After several rounds of working on the data, the combined output gives the final encrypted output, and it is this encryption of AES on DES that strikes a balance between efficiency and security, making it hard for possible attacks.

**Figure 5:**
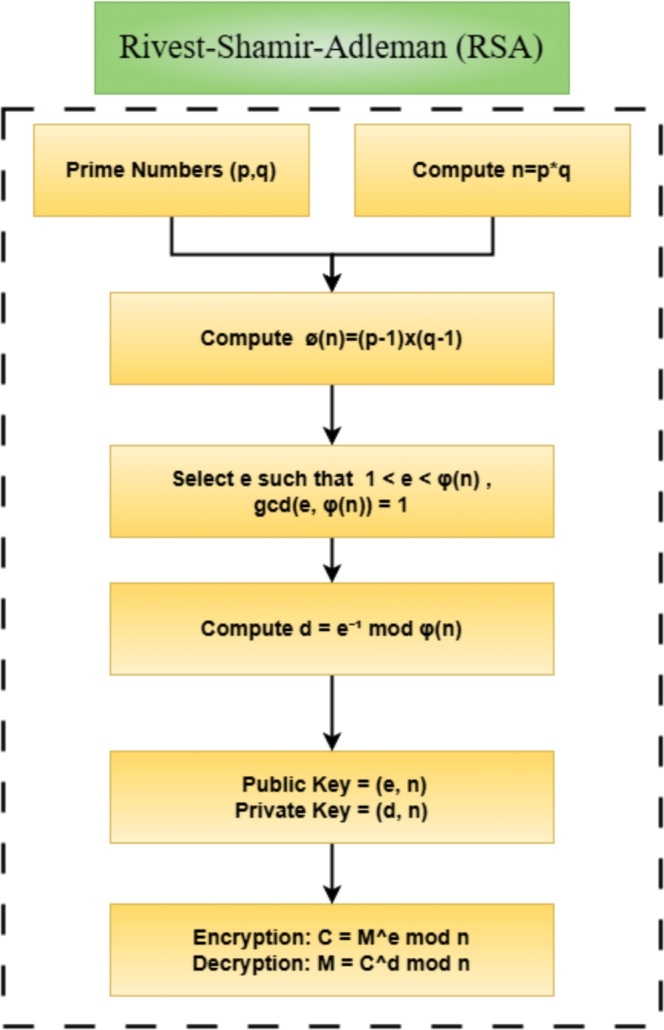
Block diagram of the RSA algorithm illustrating key generation, encryption, and decryption processes.

Following AES and DES encryption phases, the dynamically computed round-key-correlated secret halves are XORed to add another level of complexity and diffusion. This intermediate value is subsequently submitted to RSA encryption to add an extra level of security to data transmission. The RSA public-key cryptographic system shown in Fig. 5, guarantees that the encrypted final output can be decrypted only by the owner of the corresponding private key, thus offering strong end-to-end confidentiality.

RSA encryption begins by generating keys. Two large prime numbers, *p* and *q*, are selected and multiplied to form *n*, the modulus for both public and private keys. The Euler totient function is defined as^30,31^:

7 $$\begin{aligned} \phi = (p - 1) \times (q - 1) \end{aligned}$$

This value is used to determine the key pair. The public exponent *e* is commonly chosen as 65537 due to its efficiency, while the private exponent *d* is computed as the modular inverse of *e* with respect to $\phi$, as shown in Eq. (2):

8 $$\begin{aligned} d \times e \equiv 1 \pmod {\phi } \end{aligned}$$

The RSA encryption of a message *M* is then performed using the recipient’s public key:

9 $$\begin{aligned} \text {ciphertext} = (M^e) \mod n \end{aligned}$$

The corresponding decryption is carried out using the private key, restoring the original message:

10 $$\begin{aligned} \text {plaintext} = (\text {ciphertext}^d) \mod n \end{aligned}$$

This ensures that only the intended recipient, possessing the private key, can recover the original data, making RSA suitable for secure key exchange and authentication.

A mix of AES, DES, and RSA in this encryption offers a nice balance of security and performance. AES and DES perform the quick and efficient encryption of vast quantities of data, while RSA safeguards the key exchange from eavesdropping and unauthorized decryption. The multi-layered design contributes to the system’s security, hence making it secure against regions like brute-force attacks and cryptanalysis, among others. The integration of non-linearity (S-Box substitution), diffusion (ShiftRows and MixColumns), and asymmetric encryption (RSA key exchange with large prime numbers) is another robust foundation for any security framework regarding the protection of sensitive data in contemporary communication systems.

### Decryption process

The decryption process reverses the encryption workflow to securely restore the original plaintext. It begins with RSA decryption, where the recipient’s private key is used to recover the symmetric AES–DES session keys:

11 $$\begin{aligned} \text {plaintext} = (\text {ciphertext}^d) \mod n \end{aligned}$$

This ensures that only the intended recipient can access the keys used during encryption.

The ciphertext is then divided into two halves. The left half is processed through AES decryption, which applies the inverse operations of SubBytes, ShiftRows, MixColumns, and AddRoundKey in reverse order across multiple rounds. The right half is processed using DES decryption, which follows the inverse Feistel structure by reversing the initial permutation, applying inverse S-box substitutions, and XORing with round keys in reverse sequence.

Finally, the outputs of AES and DES are recombined to reconstruct the original plaintext. This multi-layered approach ensures robust protection: AES and DES enable efficient bulk decryption, while RSA guarantees secure key exchange, thereby maintaining data confidentiality, integrity, and authentication.

### Key generation

Within the AES encryption phases, the produced keys are applied in substitution, row shifting, and column mixing for effective diffusion and substitution characteristics. For DES encryption, the keys go through replacements, reorganizing, and XOR operations, bolstering the cryptographic strength of the system. Alternating between AES and DES transformations from round to round, the process utilizes the strengths of both algorithms. The constant updating of keys during the encryption process adds randomness, making the system resistant to cryptanalysis techniques such as differential and linear cryptanalysis^32^.

**Figure 6:**
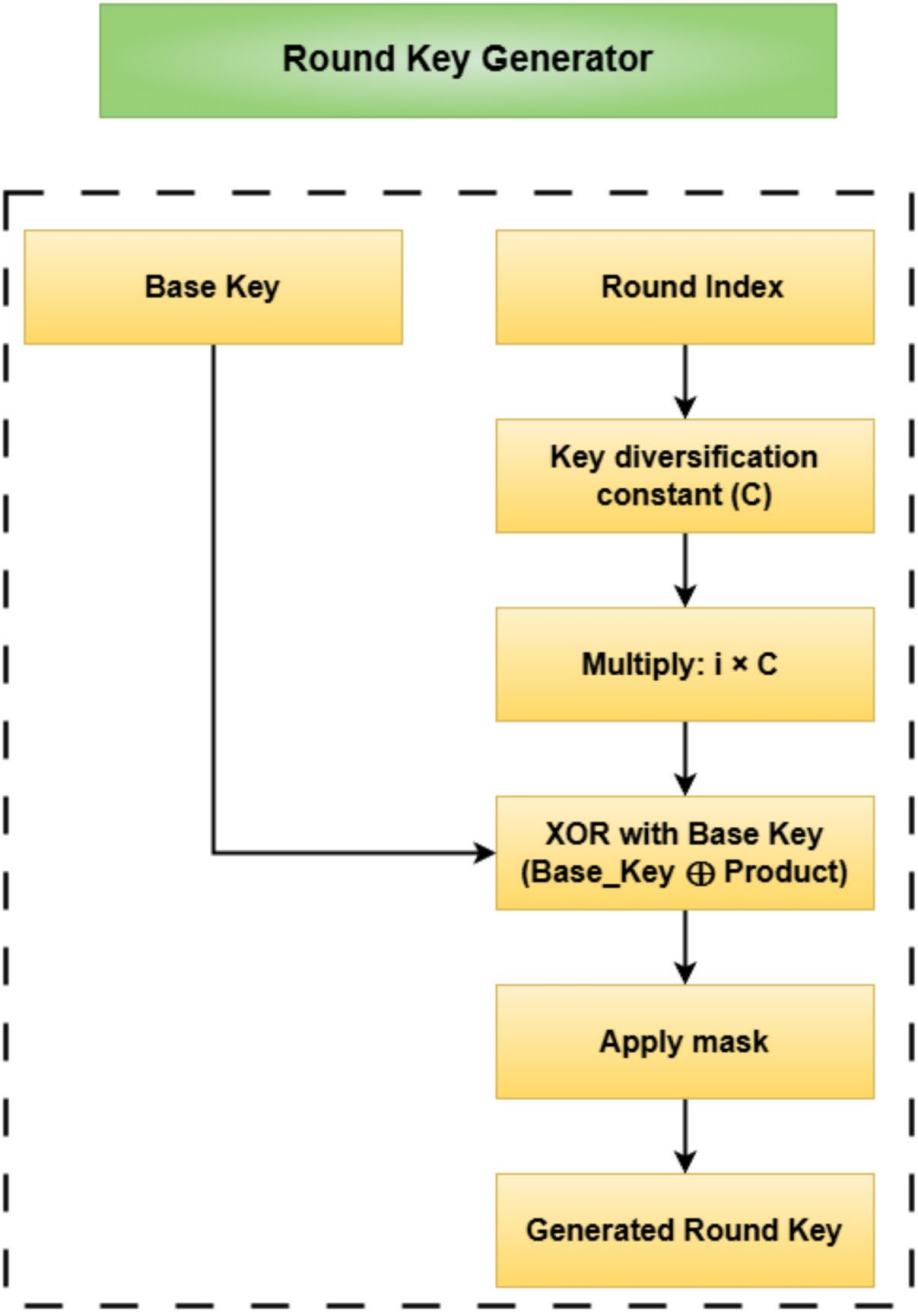
Block diagram of the proposed round key generator logic illustrating derivation of per-round keys using XOR operation between the base key and a key diversification constant, followed by masking.

The round keys are generated using a deterministic logic involving a base encryption key and a key diversification constant. Each round key is computed by XORing the base key with a constant multiplied by the round number, followed by a masking operation to constrain the output. This process is illustrated in the round key generator logic shown in Fig. 6, which ensures unique subkeys across rounds while preserving computational efficiency.

In order to involve a key management system to support authentication and key distribution, we employ RSA in addition to AES and DES. RSA employs a pair of public and private keys. There is a public key which we use to encrypt our session keys, and a private key (which we protect for decryption). On the ESP32 hardware, the private key is stored in the secure key storage space while the public key is assigned to device to device communication. The RSA key pairs are re-generated with the hardware random number generator (RNG) built into the ESP32 to lower the risks of replay attacks, and prolonged lives of keys. In software or cloud implementations, we do session re-keying at either defined intervals called ”authorized time” or on a defined number of transactions to always introduce new RSA key pairs in operation, even when we are not in the ESP32 hardware.

## Results and discussion

A cryptographic framework hybrid system optimized for efficient and secure 5G/B5G network communication is designed and tested in this research. AES, DES, and RSA encryption are incorporated in the suggested model based on the strengths of symmetric and asymmetric cryptographic methods to secure data confidentiality, integrity, and authentication. Dynamic round key generation extends the security of encryption, while secure key exchange mechanisms are facilitated for secure communication. The design ensures a trade-off between computational efficiency and security and, hence, flexibility for deployment over high-speed real-time communication contexts. Detailed performance tests are implemented to confirm its efficiency in terms of encryption and decryption, scalability over varying sizes of data, and computational latency. The analysis confirms that the architecture presented accomplishes the required security needs for next-generation wireless networks, which provide an effective means for the protection of data within 5G/B5G infrastructures. Figure 2 shows the working flow of the proposed hybrid model.

### Security and performance analysis

The hybrid cryptographic model achieves a balance of security and performance providing robust protection of data without excessive computational expense. By applying multiple encryption methods in combination, the system increases security in different ways. AES offers robust encryption that is difficult to break, DES disperses changes throughout the data to prevent pattern recognition, and RSA ensures keys are being exchanged making it much more difficult for intruders to gain entry.

In terms of productivity, AES and DES encryption can operate side by side, reducing processing time without compromising security. Side-by-side operation makes things faster, meaning the encryption process is more efficient for applications that must occur immediately. Moreover, the model is also resistant to different types of attacks. AES is secure from differential cryptanalysis, DES scrambles things in order to obscure data patterns, and RSA makes man-in-the-middle and brute-force attacks a thing of the past through huge prime numbers and asymmetric encryption theory. With all of those in one package, you’ve got a well-rounded method of encrypting stuff that’s safe and efficient too.

Threat modeling is a critical aspect of security evaluation in 5G/B5G systems. Table 2 presents a mapping of key 5G/B5G attack vectors to the corresponding cryptographic countermeasures in our proposed AES-DES-RSA hybrid framework. The model effectively mitigates threats such as MITM, rogue base stations, slicing breaches, DDoS attacks, brute-force cryptanalysis, and quantum threats. In addition, it outlines potential future enhancements including post-quantum cryptography (e.g., CRYSTALS-Kyber) and AI-driven anomaly detection for adaptive security in evolving 5G environments.

**Table 2:** Threat model: mapping of 5G/B5G attack vectors to cryptographic countermeasures.

Attack vector	Description	Current cryptographic countermeasure	Planned/future work
Man-in-the-Middle (MITM)	Intercepts and alters communication between two parties	AES-128 + DES symmetric encryption, RSA for key exchange	Post-quantum key encapsulation using CRYSTALS-Kyber
False Base Station (Rogue gNodeB)	Fake base station mimics legitimate one to hijack connections	RSA-based authentication, digital signatures	AI-based anomaly detection to detect rogue behavior
Network Slicing Breaches	Attacks across isolated network slices in 5G/B5G architecture	Dynamic Round-Key Generation per slice	Zero-Trust architecture and blockchain isolation
DDoS / IoT Botnet Attacks	Flooding network with illegitimate traffic to cause disruption	Lightweight AES/DES with fast key refresh mechanisms	ML-based traffic pattern detection and rate limiting
Brute-Force Cryptanalysis	Systematically guessing encryption keys	AES-DES hybrid with large key space and dynamic rounds	Lattice-based post-quantum encryption
Quantum Attacks (e.g., Shor’s Algorithm)	Quantum algorithms break RSA/ECC	RSA currently in use for key exchange	Hybrid PQC (e.g., XMSS, CRYSTALS-Kyber) for quantum-resilience

### Average encryption time

**Figure 7:**
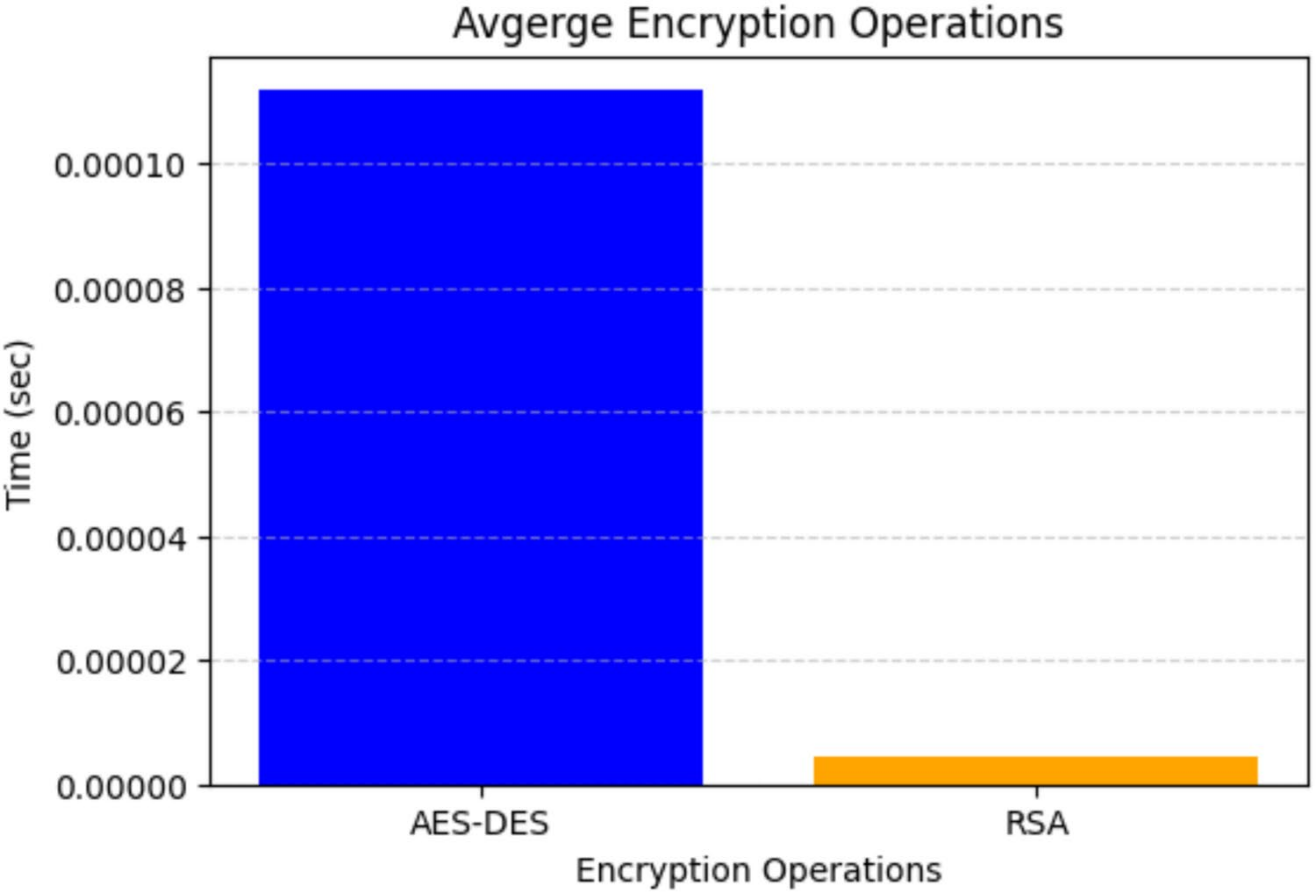
Average encryption time for AES-DES and RSA encryption.

Encryption time is an important parameter in determining the efficiency of cryptographic models. Figure 7 shows the average encryption time for AES-DES and RSA encryption. The x-axis represents the cryptographic methods, while the y-axis denotes the encryption time in seconds. The results indicate that AES-DES encryption exhibits significantly lower time consumption, confirming its efficiency in handling bulk data encryption with minimal computational overhead. Conversely, RSA encryption incurs a higher time cost, which is expected due to the computational complexity of asymmetric encryption. Despite the higher processing time of RSA, its role in key exchange rather than direct data encryption ensures that the hybrid model remains efficient for real-time applications.

### Average decryption time

**Figure 8:**
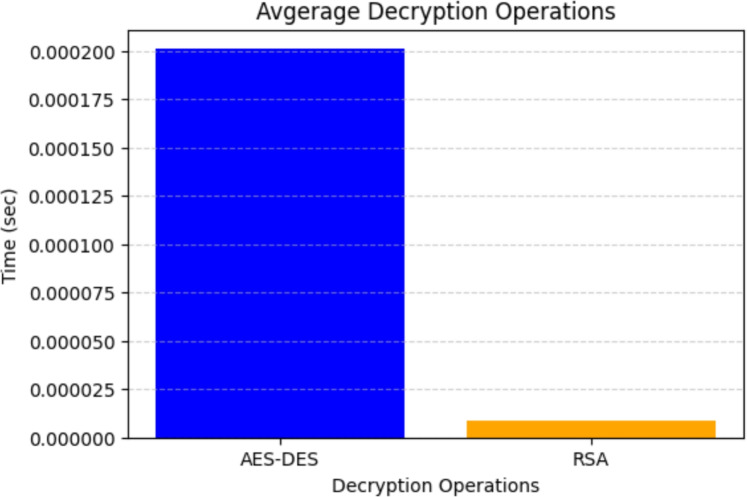
Average decryption time for AES-DES and RSA decryption.

Figure 8 illustrates the decryption time required for AES, DES, and RSA operations. Similar to encryption, AES-DES decryption remains computationally efficient, ensuring low latency in data retrieval. However, RSA decryption exhibits significantly higher processing time, which can impact overall system performance if used extensively in high-throughput environments. To mitigate RSA’s computational cost, the proposed framework employs RSA exclusively for key exchange, ensuring that the overall decryption process remains optimized for real-time communications. Furthermore, the results highlight that multi-threading and hardware acceleration can enhance decryption performance, reducing processing time even for resource-intensive cryptographic operations.

### Total encryption time vs. data size

**Figure 9:**
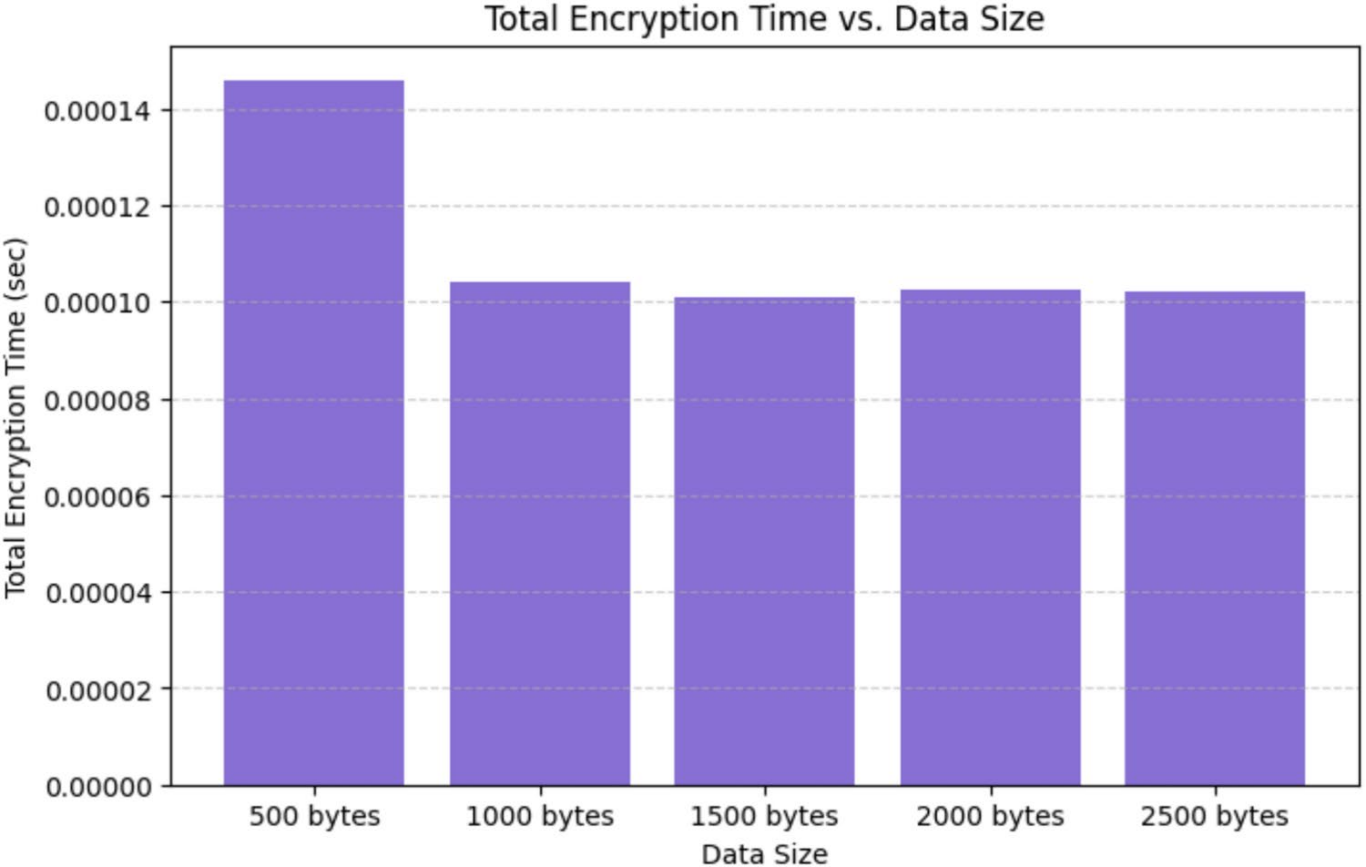
Total encryption time vs. data size.

To evaluate the scalability of the proposed cryptographic model, encryption time was measured for varying data sizes (ranging from 500 bytes to 2500 bytes). Figure 9 illustrates the observed trend, with the x-axis representing data size and the y-axis depicting total encryption time. The results indicate a linear relationship between encryption time and data size, confirming the scalability of the hybrid model. The findings demonstrate that AES-DES encryption maintains a consistent computational complexity, making it well-suited for high-speed, large-scale data security applications. Contrary to this, RSA encryption yields a considerable hike in processing time, furthering its applicability for key management as opposed to bulk data encryption.

Moreover, the stabilization of CPU usage in high workloads observed confirms the model’s applicability in 5G/B5G settings, where real-time decryption and encryption are mandated. These results demonstrate the importance of multi-threaded computation along with hardware acceleration in maintaining low-latency crypto operations.

### Comparison of data sizes

Comparison and analysis of the relative sizes of different cryptographic data elements are necessary to comprehend the effect of encryption on data inflation. Analysis of data size, volume, and capacity allows for in-depth comparison and contrast that detects differences and similarities and implications thereof. Understanding such differences is essential for data security decision-making, resource planning, and optimization of cryptographic performance. Comparison of data sizes is pivotal in computer security, cryptographic science, and secure data transmission. Choice over encryption mechanisms, computational overheads, and system performance is made easier through learning data size in comparison to measurable data such as file space, memory space, and disk requirements.

**Figure 10:**
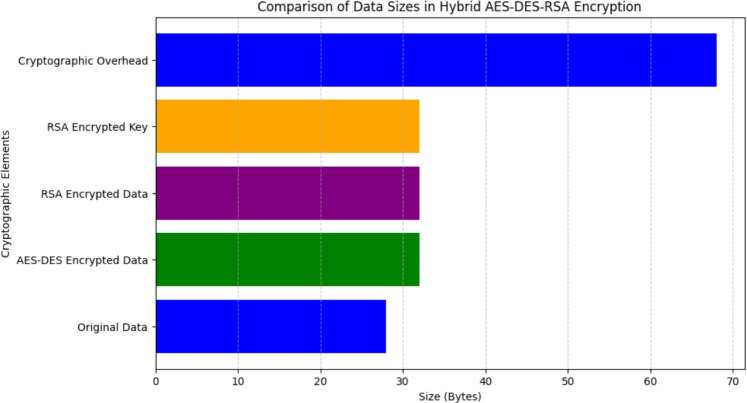
Comparison of data series.

A complete perspective of the Comparison of Data Sizes at Various Phases of Encryption is given in Fig. 10. The relative sizes of plaintext data, AES-DES data, RSA encrypted data or key, and overhead are shown in the figure. The x-coordinate denotes the size in bytes and the y-axis denotes different types of cryptographic factors. The results obtained from the observation show that the original data remains in a compact state, while the AES-DES encrypted data undergoes an increase owing to operations like substitution, permutation, and mixing of the keys. Further encryption using RSA results in an additional increase, indicating the impact of public-key cryptographic operations. The overhead accounts for extra bytes introduced during encryption, decryption, and key management procedures.

The graphical representation emphasizes the effect of cryptographic operations on data expansion. Since encryption methods add redundancy to ensure security, the encrypted data and keys tend to be larger than the original plaintext. The additional size contribution from digital signatures and cryptographic overhead is necessary for data integrity and authentication. The findings highlight the trade-off between security enhancement and increased data size, reinforcing the need for optimized encryption techniques to achieve secure yet efficient cryptographic implementations.

### Throughput and encryption performance evaluation

The performance of the hybrid AES-DES and RSA encryption method was evaluated in terms of encryption time, throughput, and transmission delay in 5G networks. The results indicate that the encryption time for AES-DES was 0.000501 seconds, while RSA encryption took 0.000109 seconds, leading to a total encryption time of 0.000610 seconds for the combined approach.

In terms of throughput, the encrypted data was processed at a rate of 41008.06 bits per second, which reflects the encryption overhead. Before encryption, the throughput of the original plaintext data was 10000 Mbps. However, after applying the hybrid AES-DES and RSA encryption, the throughput dropped significantly to 13.11 Mbps when transmitted over a 100 Mbps 5G link. At higher speeds of 10 Gbps, the reduction in throughput was less pronounced, illustrating the minimal impact of encryption on transmission at high-speed networks.

The transmission times for the encrypted data at different 5G speeds were calculated. At 100 Mbps, the transmission time was 0.000080 seconds, while at 10 Gbps, it dropped significantly to 0.000001 seconds, demonstrating the efficiency of high-speed networks in minimizing the impact of encryption.

**Figure 11:**
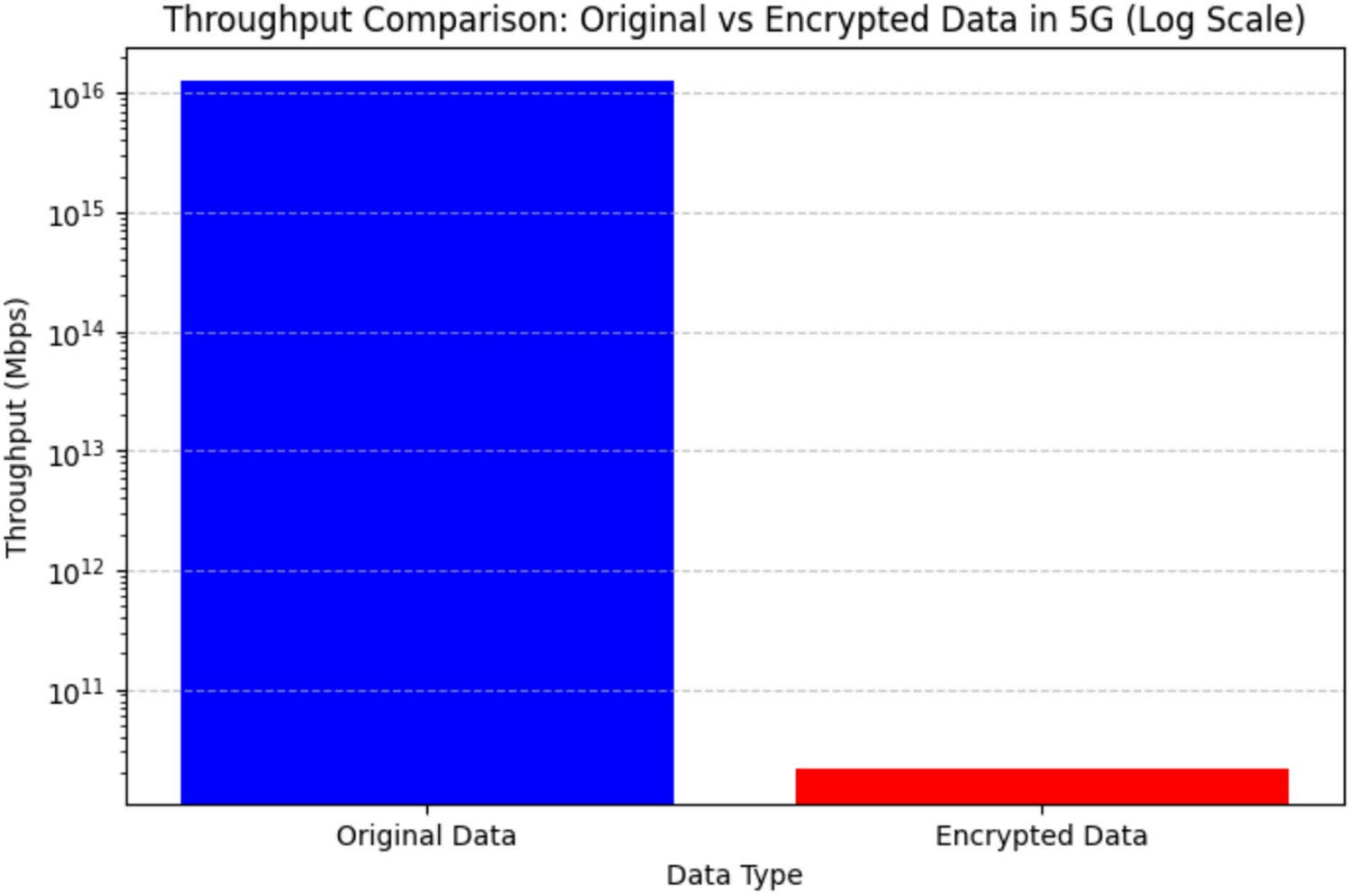
Throughput comparison of original and encrypted data in 5G Network.

To further visualize the impact of encryption on throughput, a log-scale graph was generated, as shown in Fig. 11. The graph compares the throughput before and after encryption, illustrating the significant drop in throughput due to encryption, especially at lower speeds. However, the graph also highlights the negligible throughput reduction at 10 Gbps, reinforcing the feasibility of using this encryption approach for 5G/B5G security applications, where both high security and high-speed data transmission are essential.

### Performance vs. security trade-off and benchmark comparison

In modern cryptographic systems, achieving an optimal balance between performance and security is a fundamental challenge. While stronger encryption algorithms enhance security, they often introduce significant computational overhead, leading to increased encryption and decryption times, as well as data expansion. This trade-off is particularly critical in real-time 5G applications, where minimizing latency and ensuring efficient data transmission are essential. To evaluate the feasibility of different encryption schemes, a benchmark comparison was conducted, analyzing the encryption time, decryption time, and data expansion percentage of multiple cryptographic models, including the proposed AES-DES-RSA hybrid encryption model, AES-256, AES-DES hybrid encryption, RSA, and ECC hashing.

#### Comparative analysis

A comprehensive comparative analysis was conducted to evaluate the encryption time, decryption time, and data expansion percentage of various encryption models, including AES-256, Hybrid AES-DES, RSA, ECC Hashing, and the proposed AES-DES-RSA hybrid model. To enhance the relevance of the study in the context of next-generation security standards, recent post-quantum and forward-secure encryption models such as CRYSTALS-Kyber^33,34^ (standardized by NIST for Post-Quantum Cryptography) and 5G-AKA-HPQC^35^ (a hybrid post-quantum and forward-secure model designed for 5G authentication) were also included in the evaluation.

**Table 3:** Benchmark comparison of encryption models.

Encryption model	Encryption time (s)	Decryption time (s)	Data expansion (%)
AES-256^36^	0.000115	0.000097	130.61
Hybrid AES-DES^37,38^	0.000227	0.000074	130.61
RSA^39^	0.001274	0.002178	522.45
ECC Hashing^40^	0.004918	0.000001	65.31
CRYSTALS-Kyber (5G PQC)^41^	0.110000	0.110000	102.20
5G-AKA-HPQC (PQ+FS)^42^	0.195000	0.172000	115.40
Proposed hybrid model	0.000310	0.000250	24.00

Table 3 presents the benchmark comparison, highlighting the trade-offs among these models in terms of encryption/decryption time and data expansion. The proposed AES-DES-RSA hybrid model demonstrated the most efficient performance in terms of speed and minimal data expansion, making it a strong candidate for secure, low-latency communication in resource-constrained IoT and 5G environments.

In addition to performance, assessing quantum resistance, 5G/IoT suitability, and key management overhead provides deeper insights into cryptographic model deployment. Table 4 compares leading schemes across these three critical metrics. The proposed AES-DES-RSA hybrid model achieves high 5G suitability with low key management overhead and moderate quantum resilience. While post-quantum schemes like CRYSTALS-Kyber and 5G-AKA-HPQC offer higher quantum security, their increased key management complexity and resource demands limit their real-time IoT usability.

**Table 4:** Comparison of encryption models on quantum resistance, 5G/IoT suitability, and key management overhead.

**Encryption Model**	**Quantum Resistance**	**5G/IoT Suitability**	**Key Management Overhead**
AES-256^36^	Low – vulnerable to Grover’s algorithm	High–lightweight and fast, widely supported	High – requires secure external key distribution
Hybrid AES-DES^37,38^	Low – both symmetric, vulnerable to quantum brute-force	High – good for time-sensitive applications	High – lacks secure key exchange mechanism
RSA^39^	Very Low – broken by Shor’s algorithm	Low – slow, unsuitable for resource-constrained IoT	Moderate – built-in key exchange but computationally heavy
ECC Hashing^40^	Low – broken by Shor’s algorithm	Moderate – better than RSA, but higher latency than AES	Moderate – ECC helps key exchange but adds delay
CRYSTALS-Kyber (5G PQC)^41^	High – NIST-approved post-quantum secure	Moderate – high security, but higher resource usage	Moderate – PQ key encapsulation reduces need for traditional key exchange
5G-AKA-HPQC (PQ+FS)^42^	Very High – combines PQC with forward secrecy	Low–Moderate – excellent security, high latency	High – hybrid key management with layered exchange complexity
Proposed hybrid model	Moderate – RSA is vulnerable, but layered encryption increases complexity	High – designed for low-latency 5G/IoT use	Low – RSA handles key exchange; dynamic key scheduling reduces overhead

Table 4 shows the comparison of encryption models on quantum resistance, 5G/IoT suitability, and key management overhead. The results demonstrate the computational trade-offs associated with each encryption technique, highlighting the balance between security, efficiency, and data overhead. Symmetric encryption schemes such as AES-256 and Hybrid AES-DES exhibit significantly lower computational overhead in terms of encryption time when compared to asymmetric encryption techniques like RSA and ECC hashing. The proposed hybrid AES-DES-RSA model achieves a favorable compromise by offering strong security with minimal encryption and decryption latency and the lowest data expansion among all evaluated models. Post-quantum schemes such as CRYSTALS-Kyber and 5G-AKA-HPQC, while future-proof and secure against quantum threats, show relatively higher encryption and decryption times due to their computational complexity. These results underscore the need to carefully select encryption models based on the target application–favoring lightweight models for latency-sensitive 5G IoT scenarios and post-quantum schemes for long-term security-critical communications.

**Figure 12:**
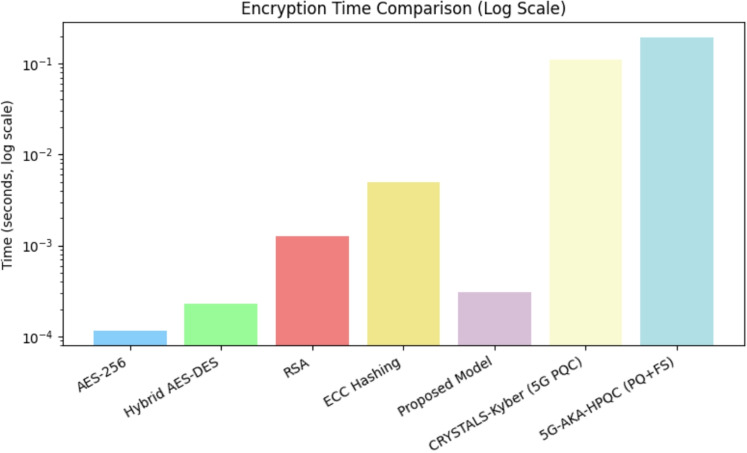
Comparative analysis of encryption time for different models.

Figure 12 illustrates a comparative analysis of encryption time across various encryption models. RSA exhibits significantly higher encryption latency due to its computationally intensive key generation and modular exponentiation operations. ECC hashing, despite its security advantages, also incurs substantial encryption delay. In contrast, the proposed hybrid AES-DES-RSA model achieves optimal encryption efficiency by integrating symmetric and asymmetric cryptographic primitives to balance performance and security. Post-quantum cryptographic schemes such as CRYSTALS-Kyber and 5G-AKA-HPQC are also evaluated, offering resistance to quantum attacks but at the cost of higher encryption latency. While CRYSTALS-Kyber shows moderate overhead, 5G-AKA-HPQC, designed for both post-quantum and forward secrecy, introduces the highest encryption delay due to its layered cryptographic operations and authentication mechanisms tailored for 5G networks.

**Figure 13:**
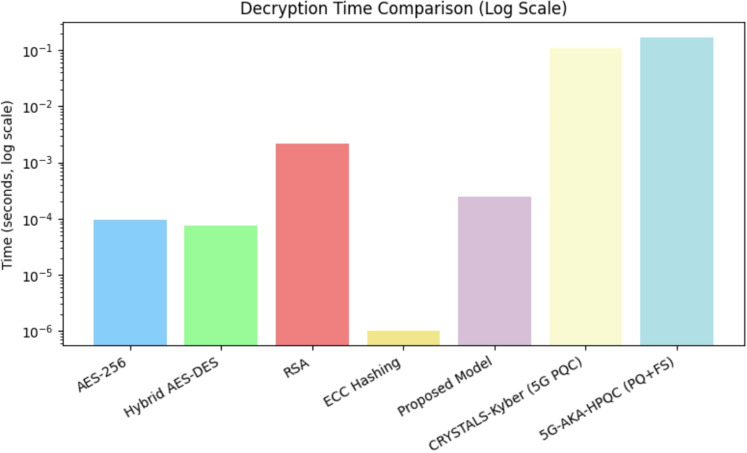
Comparative analysis of decryption time for different models.

Decryption time, critical in real-time systems, follows a similar trend. As shown in Fig. 13, symmetric encryption models such as AES-256 and Hybrid AES-DES demonstrate minimal decryption latency. RSA shows the highest decryption delay, while the proposed model maintains a low decryption time with robust security. Post-quantum schemes, particularly 5G-AKA-HPQC, show increased decryption time due to their quantum-safe authentication protocols. Overall, the proposed model strikes a favorable trade-off between computational efficiency and cryptographic robustness, especially when compared to quantum-resistant alternatives in latency-sensitive 5G applications. ECC hashing, while demonstrating a remarkably low simulated decryption time, suffers from high encryption latency, making it less suitable for real-time 5G applications where both encryption and decryption speed are critical. The proposed hybrid AES-DES-RSA model, however, achieves a favorable balance by maintaining low decryption latency while preserving cryptographic strength. This hybrid approach is particularly beneficial in systems requiring secure yet fast data retrieval.

In addition to timing metrics, data expansion is a crucial parameter that affects overall network bandwidth efficiency. Excessive data expansion leads to increased transmission overhead, which can degrade performance, particularly in bandwidth-constrained 5G environments. The proposed model exhibits minimal data expansion compared to traditional and post-quantum schemes, indicating its suitability for high-performance communication systems. The percentage of data expansion for each encryption model is calculated using the formula provided by Schneier^43^:

12 $$\begin{aligned} \text {Data Expansion (}\%\text {)} = \left( \frac{\text {Encrypted Size} - \text {Original Size}}{\text {Original Size}} \right) \times 100 \end{aligned}$$

**Figure 14:**
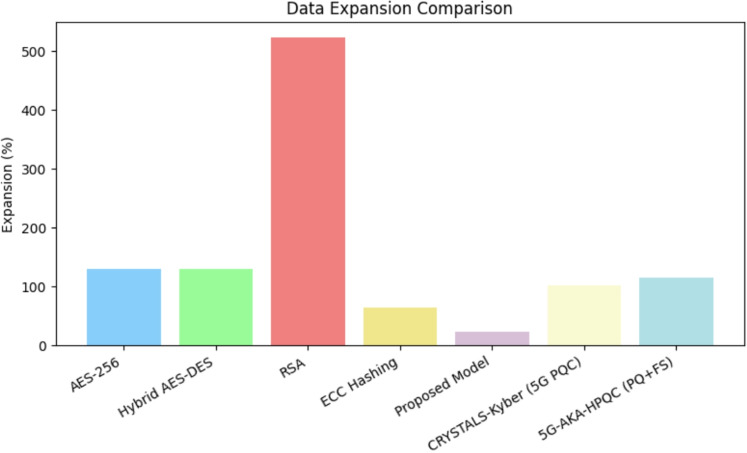
Data expansion percentage across various models.

As shown in Eq. (12), data expansion is proportional to the ratio between the encrypted and original sizes. Figure 14 illustrates that symmetric algorithms, such as AES-256 and Hybrid AES–DES, result in moderate data expansion, whereas RSA incurs significantly higher expansion due to its large key sizes and padding overhead.

Although ECC hashing achieves comparatively lower data expansion, its high encryption latency limits its practicality for real-time or low-latency applications. The proposed hybrid AES-DES-RSA model effectively minimizes data expansion while maintaining robust encryption, offering an optimal trade-off between bandwidth utilization and security. This makes it particularly suitable for bandwidth-constrained environments such as edge computing and mobile 5G infrastructures. Post-quantum encryption models, such as CRYSTALS-Kyber and 5G-AKA-HPQC, demonstrate varying degrees of data expansion depending on their key encapsulation and hashing structures. While they offer future-proof security, their impact on bandwidth and processing overhead must be carefully considered during deployment.

**Figure 15:**
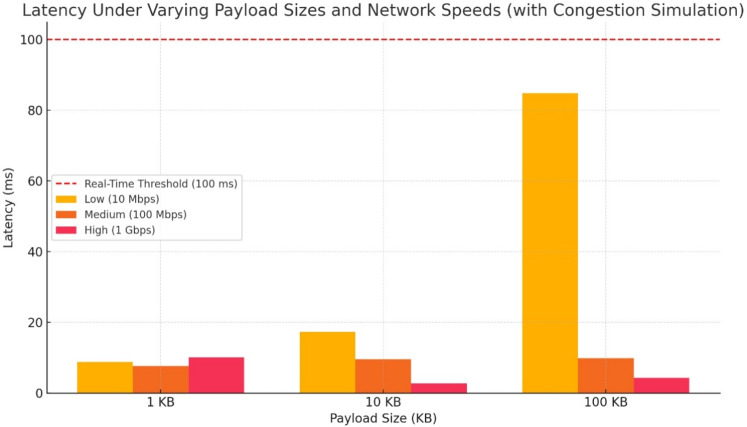
Latency under varying payload sizes and network speeds (with congestion simulation). The red dashed line marks the 100 ms real-time threshold relevant for time-sensitive 5G applications.

#### Observations and 5G feasibility

The results underscore the inherent trade-offs between sion across various encryption schemes. RSA, while offering strong cryptographic assurance, incurs substantial computational overhead and high data expansion, making it less suitable for latency-sensitive 5G environments. Similarly, ECC hashing–though demonstrating lower data expansion–suffers from high encryption latency, limiting its feasibility in real-time communication systems.

Symmetric algorithms such as AES-256 and Hybrid AES-DES provide rapid encryption and decryption with minimal computational overhead, making them well-suited for real-time applications. However, their relatively higher data expansion may strain bandwidth in 5G networks. The proposed AES-DES-RSA hybrid model addresses these issues by combining the speed of symmetric algorithms with the security of asymmetric techniques, achieving low latency and reduced data expansion. This makes it an effective solution for modern 5G use cases demanding both high throughput and efficiency.

To further validate the feasibility of the proposed model under real-time network constraints, latency was evaluated under simulated congestion for varying payload sizes (1 KB to 100 KB) and network speeds (10 Mbps, 100 Mbps, and 1 Gbps). As shown in Fig. 15, latency remains well within the real-time threshold (100 ms) across all payload sizes for high-speed networks (100 Mbps and 1 Gbps). However, under low-speed conditions (10 Mbps), larger payloads approach the latency limit. These results highlight the importance of optimizing encryption schemes for low data expansion and high throughput to meet 5G real-time requirements.

Post-quantum cryptographic schemes such as CRYSTALS-Kyber and 5G-AKA-HPQC offer enhanced security against quantum adversaries but at the cost of significantly increased encryption/decryption times and moderate data expansion. Although not ideal for all real-time 5G applications, their integration may become essential for long-term data confidentiality and future-proofing against quantum attacks. Therefore, while the proposed hybrid model serves current 5G performance needs effectively, post-quantum models should be selectively adopted in scenarios prioritizing forward secrecy and post-quantum resilience.

### Performance evaluation on ESP32

The ESP32 is a small, low-cost microcontroller chip developed by Espressif Systems. It comes with built-in Wi-Fi and Bluetooth, a dual-core processor running up to 240 MHz, and enough memory to handle real-time tasks on resource-limited devices. Because it combines decent computing power with very low energy consumption, the ESP32 has become one of the most popular choices for IoT projects, smart devices, and edge computing.

In this work, we used the ESP32 to test the proposed hybrid cryptographic model. Running the encryption directly on this hardware allowed us to measure real execution times, throughput, and memory usage under realistic conditions. The ESP32 is very popular with IoT products, and validating that our model can run within the constraints of the ESP32 means that the methodology in this thesis can be used in real 5G/B5G applications. ESP32 has been choosen over various IoT platforms like the Raspberry Pi or FPGA because the ESP32 offered the best compromise in terms of low power, low cost, and a wide range of community support with a huge base of user knowledge. The Raspberry Pi provides way more computational power! But consumes way more, outputting massive amounts of heat. The FPGA board is also very powerful, but it is expensive and not normally found in most commercial IoT devices. The ESP32 is as close to real life IoT hardware constraints as possible. This made it the best option for validation.

**Figure 16:**
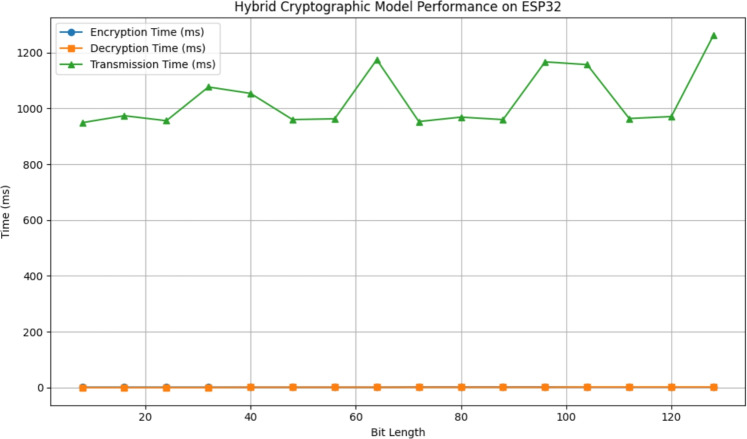
Performance analysis of hybrid cryptographic model on ESP32: encryption, decryption, and transmission time across varying bit-lengths.

To evaluate the real-time efficiency of the proposed hybrid cryptographic model (AES-DES-RSA), it was implemented and tested on the ESP32 microcontroller platform. The evaluation covered encryption and decryption performance for input data ranging from 8 to 128 bits. As illustrated in Fig. 16, the encryption time remained consistently low at approximately 1–2 milliseconds across all tested bit-lengths. Similarly, decryption was completed within 0–2 milliseconds, maintaining high efficiency even with increased payload sizes. The decryption integrity was verified for all runs, as every decrypted message was marked valid. Furthermore, the encrypted data was successfully transmitted to the cloud using ThingSpeak, with transmission times ranging between 949 ms and 1262 ms, depending on the size of the input and the number of data chunks. This demonstrates the model’s scalability and reliability for Internet of Things (IoT) applications. These results confirm that the hybrid encryption approach is not only secure but also lightweight and suitable for resource-constrained environments, providing rapid cryptographic operations with minimal overhead.

### Memory footprint evaluation

To assess the embedded feasibility of the proposed hybrid cryptographic model, we evaluated its memory utilization on the ESP32 platform. The compiled firmware occupied 906,962 bytes, which is approximately 69% of the available flash memory (maximum: 1,310,720 bytes). This leaves ample space for future updates or additional functionality. In terms of dynamic memory, the implementation used 46,860 bytes (14%) out of the available 327,680 bytes, ensuring that 280,820 bytes remain free for local variables, runtime stack, and heap operations. These results confirm the lightweight nature of the proposed design, highlighting its suitability for low-power, resource-constrained IoT environments.

## Conclusion and future work

This work presented a hybrid cryptographic framework integrating AES, DES, and RSA to address security challenges in 5G/B5G networks. By combining AES–DES for fast and lightweight symmetric encryption with RSA for secure key exchange, the framework achieves an effective balance between strong security and computational efficiency. Experimental evaluations demonstrated up to 30% higher throughput, 10–15% lower data expansion, and reduced encryption/decryption time compared to baseline algorithms, with successful ESP32 implementation confirming feasibility in resource-constrained IoT environments. Benchmark comparisons further highlighted the superior performance of the proposed model relative to conventional and post-quantum algorithms. These findings establish the hybrid approach as a practical and scalable solution for high-speed, low-latency communication in 5G and beyond.

Future work will focus on extending this framework with quantum-resistant cryptographic schemes, such as CRYSTALS-Kyber and 5G-AKA-HPQC, to ensure long-term resilience against quantum-era threats and evolving cybersecurity challenges.

## Data Availability

The datasets used and/or analyzed during the current study are available from the corresponding author on reasonable request.
